# Modulation of the Human Erythroid Plasma Membrane Calcium Pump (PMCA4b) Expression by Polymorphic Genetic Variants

**DOI:** 10.3390/membranes11080586

**Published:** 2021-07-30

**Authors:** Orsolya Mózner, Boglárka Zámbó, Balázs Sarkadi

**Affiliations:** 1 Research Centre for Natural Sciences, Institute of Enzymology, ELKH, 1117 Budapest, Hungary; mozner.orsolya@ttk.hu (O.M.); zambo.boglarka@gmail.com (B.Z.); 2 Doctoral School of Molecular Medicine, Semmelweis University, 1094 Budapest, Hungary; 3 Department of Biophysics and Radiation Biology, Semmelweis University, 1094 Budapest, Hungary

**Keywords:** plasma membrane calcium pump, PMCA4b, *ATP2B4*, erythroid cells, GATA1, SNPs, minor haplotype, dual-luciferase assay

## Abstract

In the human ATP2B4 gene, coding for the plasma membrane calcium pump PMCA4b, a minor haplotype results in the decreased expression of this membrane protein in erythroid cells. The presence of this haplotype and the consequently reduced PMCA4b expression have been suggested to affect red blood cell hydration and malaria susceptibility. By using dual-luciferase reporter assays, we have localized the erythroid-specific regulatory region within the haplotype of the ATP2B4 gene, containing predicted GATA1 binding sites that are affected by SNPs in the minor haplotype. Our results show that, in human erythroid cells, the regulation of ATP2B4 gene expression is significantly affected by GATA1 expression, and we document the role of specific SNPs involved in predicted GATA1 binding. Our findings provide a mechanistic explanation at the molecular level for the reduced erythroid-specific PMCA4b expression in carriers of ATP2B4 gene polymorphic variants.

## 1. Introduction

The human plasma membrane Ca^2+^ ATPase 4b (PMCA4b), encoded by the *ATP2B4* gene on chromosome 1, functions as an ATP-driven Ca^2+^ pump and is responsible for numerous cellular functions, including the removal of excess cytoplasmic calcium and participating in calcium-dependent cellular signaling (for reviews, see [1,2,3]). The PMCA4b protein is particularly responsible for the low calcium levels in cells of hematopoietic origin, including platelets and erythrocytes, but also participates in calcium regulatory functions in many other cell types, including neurons [3,4,5]. Genome-wide association (GWA) studies have indicated that a given set of single nucleotide polymorphisms (SNPs) in *ATP2B4* confers resistance to a severe form of malaria among children, protects against malaria and associated maternal anemia, and modulates cell dehydration [6,7]. Moreover, a specific missense mutation in this gene was shown to result in dominant familial spastic paraplegia, based on reduced PMCA4b function in specific neurons [8].

In 2017, two independent studies examined the role of an *ATP2B4* minor haplotype in modulating the expression and regulation of PMCA4b in erythroid cells. This relatively large haplotype resides in the region of intron 1 and exon 2, a predicted promoter/enhancer area of the *ATP2B4* gene. Zámbó et al. [9] found that individuals carrying the minor haplotype show significantly decreased PMCA4b expression in the red blood cell membrane. PMCA4b expression in the erythrocytes of individuals homozygous for this minor haplotype was reduced by almost 50%, correlating with a functional deficiency of active calcium extrusion. In a detailed study, Lessard et al. [10] found that the same minor haplotype caused decreased PMCA4b protein expression specifically in erythroid cells. By using a CRISPR/Cas-9 genetic engineering method, they generated stable HUDEP-2 (erythroid progenitor) and HEK293 cell lines in which an intronic region before the 2nd exon (within this haplotype) was deleted. The lack of this intronic enhancer region resulted in a major reduction in PMCA4b mRNA expression and increased calcium levels in the HUDEP-2 cells, while there was no change in PMCA4b expression in the non-erythroid type HEK cells.

The results of Zámbó et al. [9] and the comprehensive study of Lessard et al. [10], performed in human erythroid and erythroid progenitor cells, as well as using a rat knock-out model, clearly documented the presence of an erythroid-specific enhancer region in the *ATP2B4* gene, causing a major effect on PMCA4b expression. Although these experiments indicated the potential role of some transcription factors, the mode of regulation and the transcription factor(s) involved have not been identified so far. Analyzing the changes in transcription binding sites in the minor haplotype of this enhancer, a potential GATA1 binding site has been found to be altered [11,12]. The GATA1 protein (along with GATA2) is the master regulator of erythroid lineage maturation, including erythropoiesis, and a large-scale GWA study of the Malaria Genomic Epidemiology Network [13] reinforced the role of these *ATP2B4* gene regions and their potential GATA1 regulation in malaria susceptibility. The *ATP2B4* gene polymorphism has been suggested to be a malaria-driven selection force in some African regions [14]. Therefore, in the present work, we performed a study to examine the molecular background and the SNPs involved in the potential erythroid-specific regulation of PMCA4b expression.

## 2. Materials and Methods

### 2.1. Vector Constucts

Different regions of the ATP2B4 gene (see Supplementary Figure S1) were cloned into pGL3 Firefly luciferase plasmids without additional upstream promoter sequence by using standard restriction enzyme-based cloning. Sequences were amplified from DNA extracted from samples carrying the WT or the haplotype in homozygous form [9].

Site-directed mutagenesis on the H1st WT-containing plasmid to 4945, 51 or 4945/51 was carried out by standard mutagenesis protocol using Platinum SuperFi polymerase (Thermo Fisher 12351010, Waltham, MA, USA) and the following primers:
4945mut_for5′ CACCTTCAGCCCTCCGTTTTGTCACCTACACCACACC 3′4945mut_rev5′ GGTGTGGTGTAGGTGACAAAACGGAGGGCTGAAGGTG 3′51mut_for5′ GAGAGGTATCTTACCGCTCCCACTCCAG 3′51mut_rev5′ CTGGAGTGGGAGCGGTAAGATACCTCTC 3′

For GATA1 co-expression experiments, GATA1 coding cDNA was cloned into the pIRES2 expression vector that contains a CMV promoter and enhancer sequence, along with a GFP coding sequence, independently translated from GATA1, as transfection control.

### 2.2. Transfection of HEK, HEL and K562 Cells

HEL cells were cultured in T25 vented cap cell culture flasks in RPMI-1640 cell culture medium, K562 cells in IMDM cell culture medium, both supplemented with 10% FBS, 100× GlutaMAX and 100× Pen/Strep. Prior to transfection, K562 and HEL cells were seeded onto a 96-well cell culture plate at 2.5 × 10^4^ cells/well, in 80 µL Opti-MEM serum-free cell culture medium. A total of 0.2 µL Mirus TransIT 2020 transfection reagent and 100 ng total plasmid DNA/well were used for transfection. A 10:1 molar ratio of the examined vector (pGl4-Firefly) and co-reporter (pRL-TK-Renilla) vector was used. The transfection reagent and plasmid DNA were diluted in Opti-MEM. Five hours after transfection, the Opti-MEM cell culture media were supplemented with 200 µL fresh complemented appropriate medium. HEK293 cells were cultured in DMEM medium supplemented with 10% FBS, 100× GlutaMAX and 100× Pen/Strep. HEK 293 cells were transfected with Lipofectamine 2000 reagent in Opti-MEM, and the same amounts and methods were used as described above with the following differences: one day before transfection, HEK293 cells were plated on 96-well cell culture plates, and 5 h after transfection, the transfection mix diluted in Opti-MEM was replaced with fresh medium.

For GATA1 co-expression experiments, transfection was performed as described above. A 7:3:1 molar ratio of the (pGL4-Firefly:pIRES2-GATA1:pRL-TK-Renilla) vectors was used. In the control (no GATA1) experiments, the pIRES2 empty plasmid was transfected along with pGL4 and pRL-TK plasmids.

### 2.3. Dual-Luciferase Reporter Assay System

The dual-luciferase reporter assay system was used according to the manufacturer’s guidelines for 96-well cultured cells (Promega Dual-Luciferase Reporter Assay system E1910 Instructions). Results were read by a VictorX3 Multilabel Plate Reader. Reagents were added on 96-well white plates to the cell lysates manually, immediately before reading the signals one by one. Firefly luminescence was read after mixing 20 µL of cell lysate and 50 µL LARII reagent, Renilla luminescence was read after the addition of 50 µL Stop & Glo reagent. At least two technical and two biological parallel experiments were performed in all cases. The data were normalized to HEK WT (and HEK WT minus GATA1—the detailed data and normalizations are shown in Supplementary Table S1). For statistical analyses, two-tailed unpaired Student’s *t*-tests were performed.

### 2.4. RNA Isolation, cDNA PCR

RNA isolation was performed by the PureLink RNA Mini Kit according to the manufacturer’s protocol from 1 × 10^6^ cells harvested for 3 days prior to RNA isolation. High-Capacity cDNA Reverse Transcription Kit (Applied Biosystems Cat. 4368814, Waltham, MA, USA) was used for reverse transcription. GATA1 specific primers were used to amplify GATA1 cDNA in a PCR reaction with Phusion HF DNA polymerase.

### 2.5. Western Blotting

Proteins were isolated from HEK293, HEL92 and K562 cells in TE sample buffer (0.1 M TRIS-PO4, 4% SDS, 4 mM Na-EDTA, 40% glycerol, 0.04% β-mercaptoethanol and 0.04% bromophenol blue) for Western blot experiments. For detecting the respective proteins, the anti-GATA1 (Abcam ab181544) rabbit monoclonal and anti-β-actin (Sigma, cat. A1978, St. Louis, MO, USA) mouse monoclonal primary antibodies, goat anti-mouse IgG (H + L) HRP conjugate (Abcam ab97023, Cambridge, UK) and goat anti-rabbit IgG (H + L) HRP conjugate (Abcam ab6721) secondary antibodies and Pierce™ ECL Western Blotting Substrate were used.

## 3. Results

In these experiments, we examined the promoter activities of the first part of the *ATP2B4* haplotype region, which has been shown to have the highest regulatory potential at transcription level. This region includes several transcription binding sites and was shown to have a pronounced effect on transcription [10,12]. To assess the transcription binding sites important in regulation, firstly we needed to identify the narrower regulatory unit within the haplotype.

**Figure 1:**
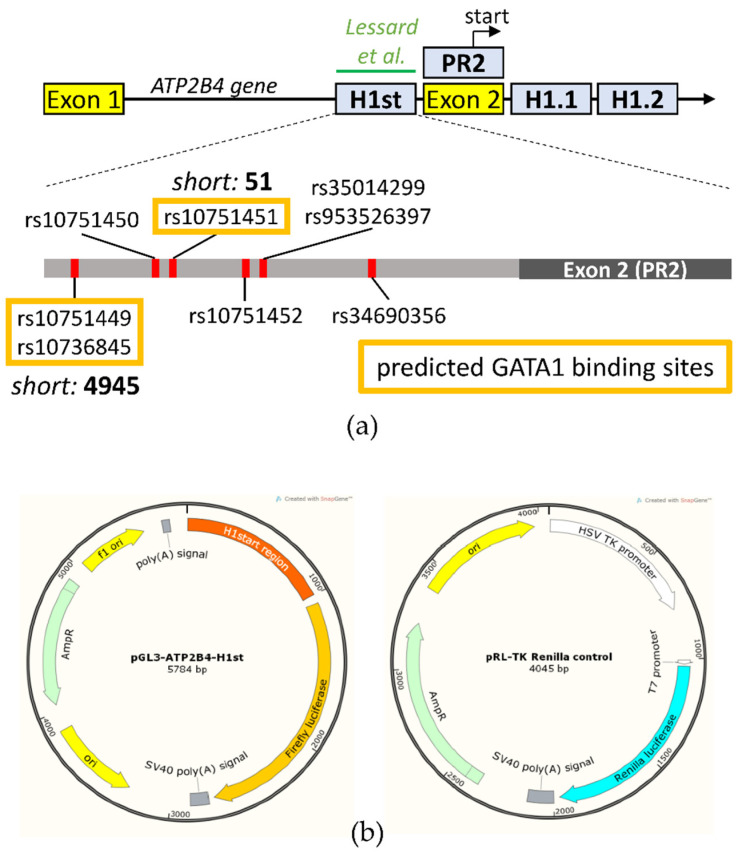
(**a**) Schematic presentation of haplotype location in the *ATP2B4* gene, previously indicated to be responsible for the regulation of PMCA4b expression. Yellow boxes label the first and the second exon—the promiscuous promoter is located around exon 1, while the recently identified enhancer/second promoter is located around exon 2, where the haplotype can be found. Blue boxes indicate the constructs used in this study. In the H1-start (H1st) region, corresponding to the erythroid-specific regulatory region described by Lessard et al. [10] (green), the SNPs of the minor haplotype are also depicted. SNPs predicted to eliminate GATA1 binding sites are labeled by yellow boxes. (**b**) Maps of plasmids used in the dual-luciferase expression assays (created by SnapGene).

As shown in Figure 1a, we examined four regions, which were the haplotype 1 start region (H1st, previously documented to be an erythroid-specific enhancer by Lessard et al. [10]), the potential core of a second promoter region (PR2) within exon 2, and the haplotype 1.1 and 1.2 (H1.1 and H1.2) regions, both downstream of exon 2, all of which involve SNPs of the haplotype and are potentially important in the regulation of transcription. Figure 1a is a schematic presentation of the haplotype in the *ATP2B4* gene, previously indicated to be responsible for the regulation of PMCA4b expression. The enlarged area depicts the SNPs present in the potential erythroid-specific enhancer, the H1st region.

In order to clarify the role of these regions, we performed preliminary dual-luciferase expression experiments, in which the three different cell lines used were human embryonic kidney HEK293 cells and two erythroid cell lines, HEL92.1.7 (erythroleukemia) and K562 (from chronic myelogenous leukemia, hematopoietic progenitors of the erythrocyte, granulocyte and monocytic series). These cells were transfected with Firefly luciferase-based reporter DNA plasmid constructs containing (without an artificial promoter) the putative promoter/enhancer regions from *ATP2B4* (see Figure 1b left). The co-transfected control plasmids contained Renilla luciferase cDNA, its stable expression driven by a thymidine kinase promoter (Figure 1b right). For measuring the expression/activity of Firefly and Renilla luciferase, the substrates were luciferin and coelenterazine, respectively. By using this dual-luciferase assay and calculating the ratio of Firefly/Renilla (F/R) luciferase activities, the potential differences in the transfection efficiency in the various cell lines were corrected (see Section 2).

In our preliminary studies, when the Firefly luciferase expression was driven by the combined sequences of the H1st plus the PR2 regions, a major activation of the luciferase expression (an F/R ratio of 200–300 (see Supplementary Figure S2)) was observed in the HEK cells, while much less expression activation was found in the HEL and K562 cells (F/R ratios between 10 and 20). These expression levels were not different when the wild-type (WT) or the minor haplotype (H) sequence was inserted into the luciferase expression vector. Similar F/R activity ratios were obtained if the constructs contained either the WT or the haplotype (H) version of the PR2 cDNA regions alone, thus the PR2 region indeed behaved as a promoter for Firefly luciferase expression, with high activity in the HEK cells and with less intensity in the HEL and K562 cells.

**Figure 2:**
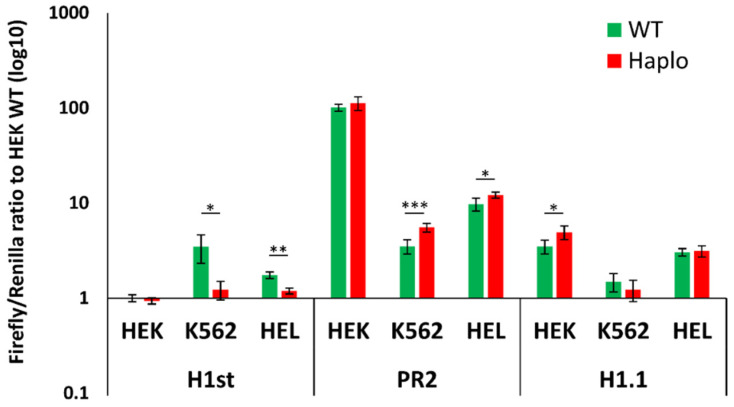
Evaluation of the dual-luciferase assay results by comparing the wild-type (WT) and the minor haplotype region (Haplo)-driven luciferase expression. The Firefly/Renilla luciferase activity ratio was normalized to the activity obtained in HEK cells with the WT constructs. Replicates: *n* = 5 in case of HEK and K562, *n* = 4 in case of HEL cells (significance by *t*-test: * *p* < 0.05, ** *p* < 0.01, *** *p* < 0.001).

Figure 2 depicts the above luciferase expression results by normalizing the F/R ratios obtained in the haplotype constructs to those measured in the constructs containing the WT forms of the selected *ATP2B4* regions in HEK cells. When the WT form of the H1-start cDNA was inserted into the Firefly luciferase vector, very low expression was found in the HEK cells. In contrast, in the K562 and HEL cells, higher luciferase expression was observed (F/R ratios higher, around 5 or 2), while in these cells the expression driven by the minor haplotype sequence (H1-start minor H) was much lower, practically indistinguishable from that in the HEK cells (F/R ratio around 3). Interestingly, when the PR2 site was inserted in front of the luciferase gene, we saw an immense expression from this construct in HEK cells, while in K562 and HEL cells the expression was much lower. In erythroid cells, higher expressions from the haplotype-containing construct could be observed. The luciferase expression from H1.1 was somewhat higher in HEK cells from the haplotype-containing vector than from WT. In case of the H1.2 region, we did not observe any promoter activity (data not shown), which may indicate the margin of the promoter region. Therefore, this region was excluded from our further experiments.

In the following experiments, we focused on the potential role of the H1-start region by further examining the role of the six SNPs in the H1-start region of *ATP2B4* (see Figure 1a) in causing the differences in luciferase expression in the erythroid cell lines. Since three SNPs (rs107751449, rs10736845 and rs10751451) in this region affect a predicted GATA1 transcription factor binding position (Alggen Promo [11,12]), first we examined the potential role of GATA1 expression in the examined cell types.

**Figure 3:**
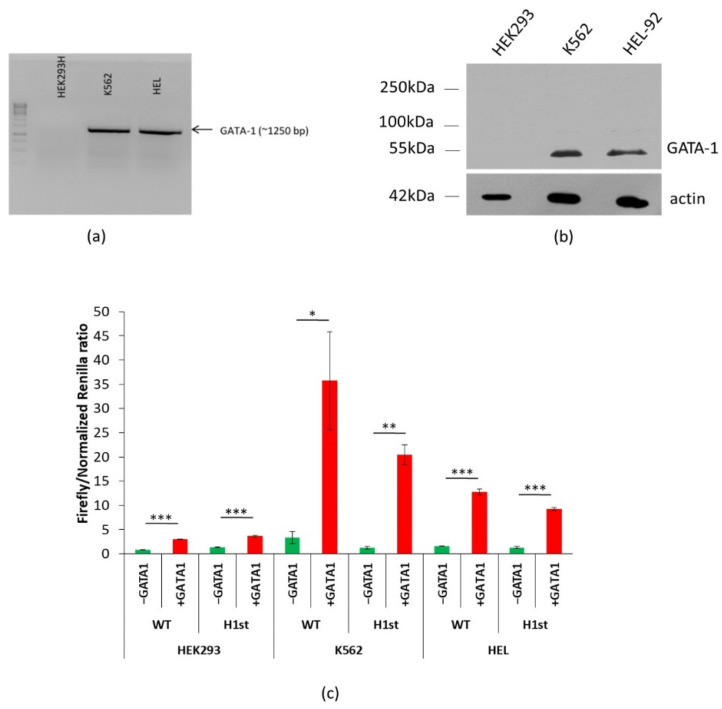
Potential role of GATA1 in the regulation of PMCA4b expression. For details, see Section 2. (**a**) GATA1 mRNA expression in HEK, K562 and HEL cells. (**b**) GATA1 protein expression HEK, K562 and HEL cells—Western blot of total protein extractions from HEK293, K562 and HEL-92 cells with anti-GATA1 and anti-actin primary, HRP-labeled secondary antibodies. (**c**) Dual-luciferase assay in the GATA1-expressing, transfected cells. The inserted WT or the minor haplotype (H1) versions of the *ATP2B4* regions were expressed in HEK, K562 and HEL cells along with pIRES2-GATA1 or pIRES2 empty vector. Normalization of the Renilla luciferase activity in the case of GATA1 overexpression was used, see details in Supplementary Table S1. The ratio of the Firefly/normalized Renilla luciferase activity is shown (*n* = 4, unpaired two-tailed *t*-test: * *p* < 0.05, ** *p* < 0.01, *** *p* < 0.001).

To validate that GATA1 is only expressed in the examined erythroid cell lineages, first we verified GATA1 mRNA levels by reverse transcription PCR (RT-PCR) in the cell lines used for the above experiments. As shown in Figure 3a, we did not detect GATA1 mRNA in HEK cells, while this expression was clearly detectable in the K562 and HEL cells. To further validate the lack of GATA1 protein in these cells, we performed Western blots with anti-GATA1 antibody (see Section 2), and we did not see any GATA1 expression in HEK293 cells, while in K562 and HEL cells the protein was clearly observable (Figure 3b). Thus, this expression pattern may support the notion that GATA1 could be a main transcription factor involved in the enhancement of PMCA4b expression in erythroid cells.

In order to further establish the role of GATA1 in the enhancer activity, we transfected the HEK, K562 and HEL cells with an expression vector containing the GATA1 coding cDNA, or with an empty pIRES2 vector as a control, and performed the dual-luciferase assay in these GATA1-overexpressing cells (Figure 3c). In these experiments, the luciferase expression constructs contained either the WT or the minor variant of the H1-start sequence of the *ATP2B4* gene. In the case of the HEK cells, GATA1 overexpression only slightly increased the luciferase expression, both in the case of the WT and the variant H1 constructs. In contrast, GATA1 overexpression greatly increased luciferase expression in the K652 and HEL erythroid cells compared to the control vector lacking the *GATA1* sequence. High luciferase expression by GATA1 co-expression in the erythroid cell lines was more pronounced from the plasmid containing the wild-type H1-start than that containing the minor variant of this region.

In further experiments, we focused on the actual role of the SNPs in the predicted GATA1 transcription factor binding sites within the H1-start region. Therefore, we selectively mutated the SNPs (rs107751449, rs10736845 and rs10751451) in the predicted GATA1 transcription factor binding sites. We generated luciferase expression constructs containing the minor variant SNPs, rs107751449 + rs10736845 (shortly labeled as 4945, as in Figure 1), and constructs containing only the SNP rs10751451 (labeled as 51 in Figure 1). We also generated luciferase expression constructs containing the combined three minor variant SNPs, rs107751449, rs10736845 and rs10751451 (labeled as 4945/51), in this vector. We measured the promoter activity of these constructs without GATA1 overexpression in K562 cells.

**Figure 4:**
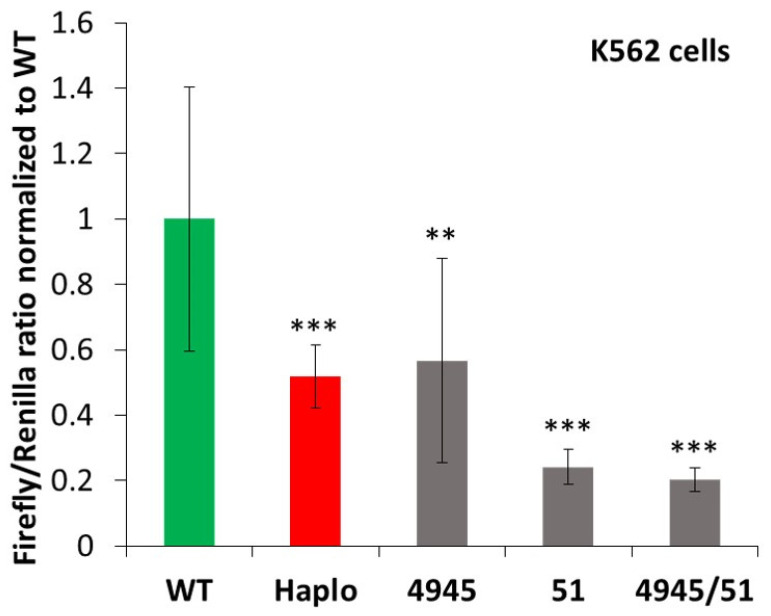
The role of selected SNPs in modulating Firefly luciferase expression in the dual-luciferase assay, performed in K562 erythroid cells. Selected SNPs potentially affecting GATA1 binding were mutated to the haplotype sequence and the promoter activities were measured. The ratio of the Firefly/Renilla luciferase activity normalized to WT is shown. See text for further details. Stars: significance by unpaired two-tailed *t*-test, compared to WT (** *p* < 0.01, *** *p* < 0.001), *n* = 6.

As shown in Figure 4, when expressing these constructs in K562 cells, we found that the expression of the full minor haplotype strongly reduced luciferase expression (see also Figure 2). A similar reduction was observed in the case of the construct containing the two SNPs predicted in one of the GATA1 binding regions (4945, rs107751449 + rs10736845). Interestingly, construct 51 (containing only the minor variant of SNP, rs10751451), also predicted to be involved in GATA1 binding, caused an even larger reduction in luciferase expression, and the construct containing all three SNPs (4945/51), similarly to 51, greatly reduced luciferase expression.

## 4. Discussion

The role of *ATP2B4* gene polymorphisms, as one of the potentially important factors in malaria susceptibility, has recently attracted much attention and initiated both experimental and computational studies (see references below). Based on these studies, it has been established that an erythroid-specific regulatory region of the *ATP2B4* gene is present in the first intron region, and SNPs located in this region significantly affect both PMCA4b expression and susceptibility to malaria. The malaria-protective SNPs in *ATP2B4* also associate with increased mean corpuscular hemoglobin concentration (MCHC) in the red blood cells, and thus may cause a wider disease-connected phenotype. The mechanism through which reduced PMCA4b expression affects parasitic disease is currently unknown—the changes in cellular calcium homeostasis or pleiotropic effects of this calcium pump expression have been suggested [9,10,13].

The regulatory region in *ATP2B4* (see Figure 1) has been explored and the role of GATA1 has been indicated in several publications [10,14,15]. However, the exact role of specific SNPs in this erythroid-specific expression regulation has not been established. The results presented here should provide a better understanding of the regulation of the *ATP2B4* gene by the SNPs within the predicted (H1-start) enhancer region, and the modulation of the enhancer properties of this sequence by naturally occurring SNPs.

In this work, we have generated luciferase expression-based reporter constructs and examined the effects of SNPs in human non-erythroid and erythroid model cell lines. The proposed *ATP2B4* promoter/enhancer regions were inserted into a promoterless expression plasmid and luciferase expression (corrected for Renilla luciferase expression, driven by a minimal promoter) was examined. According to our results, the PR2 (exon 2) region is highly active in HEK293 cells, while it has much lower promoter/enhancer activity in the examined erythroid cell lines (K562, HEL). In the erythroid cells, somewhat higher PR2 activity could be observed in the case of the minor haplotype construct. The H1.1 region also showed promoter/enhancer activity, and in this case only a minor increase in the luciferase expression was observed in the presence of the minor haplotype construct, and only in HEK cells.

The H1-start region—which was indicated to have a major role in gene regulation in erythroid cells—had promoter/enhancer activity, and this activity in the non-erythroid HEK cells was very low and not altered by the presence of the minor variant SNPs. In contrast, in the erythroid cell lines, the minor variant had significantly reduced promoter/enhancer activity as compared to the wild-type sequence. In addition, the overexpression of GATA1 increased the promoter/enhancer activity of the examined regions in HEK293 cells independently of the presence or absence of SNPs, while this effect of GATA1 was significantly more pronounced in the erythroid cell lines and strongly reduced in the presence of the minor variant of the H1-start region. These results suggest that this region harbors an erythroid-specific transcription factor binding site—most probably a GATA1 binding site, which is diminished in the minor haplotype.

In order to further explore the role of specific sequences, we selectively mutated the SNPs predicted to be involved in GATA1 binding within the H1-start region and expressed the luciferase-based reporter construct in the erythroid K562 cell line. These experiments indicated that the two predicted GATA1 binding sites, eliminated by the presence of minor SNPs, are both functionally important in erythroid cells. The luciferase expression constructs containing these affected minor SNPs, that is, rs107751449 + rs10736845 or rs10751451, had significantly reduced promoter/enhancer activity. Interestingly, the minor variant of rs10751451 had a more pronounced effect on this activity, either used separately or together with the other two SNPs.

The results showing a stronger effect of the selected SNPs than the whole-length H1-start region on protein expression may point to the exact molecular background of the polymorphic variants affecting malaria susceptibility or other blood-related conditions. Interestingly, a similar role of a polymorphic GATA1 binding site in the promoter region has been documented in modulating the expression of the Duffy blood group antigen, a receptor for Plasmodium in erythroid cells, thus reducing malaria infection [16]. In our work, because of technical limitations, we could not analyze the large promoter/enhancer regions of *ATP2B4*, thus the entire promoter region, including the first promoter upstream of exon 1, may have additional complex regulatory properties.

While our results document the functional role of the specific haplotype region and SNPs in GATA1 regulation of *ATP2B4* expression in erythroid cells, these results also suggest that, in addition to GATA1, other transcription factors may also be involved in the erythroid-specific regulation of this enhancer region. The potential complex role of regulatory factors is indicated by the relatively small effect of GATA1 expression in the HEK cells compared to that in the erythroid cell lines, as these latter cells may also contain additional, erythroid-specific transcription factors. Additionally, in our experiments (not shown in detail here), we failed to find a direct GATA1 protein binding to the H1-start DNA sequences by electrophoretic mobility shift assay (EMSA). Therefore, additional studies are required to clarify the role of GATA1 and/or other complexing factors in the regulation of this enhancer region.

The exact role of the PMCA4b variants is still unclear in malaria, as other PMCA isoforms may have a compensatory role. However, a recent study searching for the genetic background of malaria susceptibility reported the generation of human induced pluripotent stem cells with knocked-out Basigin or *ATP2B4* genes (Pance et al., 2021, preprint [17]) and demonstrated the altered susceptibility of the differentiated erythroid cells to Plasmodium infection in both cases. Another current study in mice with systemic knock-out of PMCA4 [18] indicated that in these KO mice, malaria infection has a more pronounced central nervous system effect. In further advanced molecular genetic studies, the modulation of the exact *ATP2B4* regulatory elements reported here may significantly help to perform targeted gene-editing for exploring disease-modulating polymorphisms.

## 5. Conclusions

The results presented here point to the importance of a limited promoter/enhancer region and demonstrate the functional role of GATA1 in modulating the expression of the *ATP2B4* gene in polymorphic genetic variants. These results also depict the corresponding SNPs involved in this regulation and suggest a mechanism for decreased red blood cell PMCA4b expression observed among the carriers of the minor haplotype of the *ATP2B4* gene, connected to decreased malaria susceptibility.

## Data Availability

All data presented in the manuscript are included in Supplementary Table S1.

## Supplementary Materials

The following are available online at https://www.mdpi.com/article/10.3390/membranes11080586/s1, Figure S1: DNA sequences inserted into pGL4-Firefly luciferase plasmid. Location of SNPs of the haplotype. Figure S2: Dual-luciferase measurements with the examined H1st, H1.1, PR2, H1st–PR2 constructs in HEK, HEL92, K562 cells, data are shown before normalization to HEK WT. Table S1: All dual-luciferase raw data, normalization and statistical analyses.
