# Supplementary material for: Modulation of the Human Erythroid Plasma Membrane Calcium Pump (PMCA4b) Expression by Polymorphic Genetic Variants
Source: Membranes (Basel). 2021 Jul 30;11(8):586. doi: 10.3390/membranes11080586 (PMC8401972; doi:10.3390/membranes11080586)
Supplement: Supplementary file 1 [file membranes-11-00586-s001.zip › membranes-1314708-supplementary.pdf]

membranes

MDPI

Article

Modulation of the human erythroid plasma membrane calcium pump (PMCA4b) expression by polymorphic genetic variants

Orsolya Mózner [1 ¶], Boglárka Zámbó [2,¶,†] and Balázs Sarkadi [1,2,*]

[1] Institute of Enzymology, Research Centre for Natural Sciences, ELKH, Budapest, Hungary
[2] Department of Biophysics and Radiation Biology, Semmelweis University, Budapest, Hungary
¶ These authors contributed equally to this work.
† Current address of B.Z.: Institute of Genetics and Molecular and Cellular Biology (IGBMC), Illkirch, France
* Corresponding author: sarkadi@biomembrane.hu

H1-start (H1st):

CCCCACTCTGAACTGAGAAGGGGCCTCAGGATAAGTTCTCTTCCCACCTTCAGCCCTCCGTATCGTCACCTACACCACACCCCCTAGTTAGCAT
GCGTGAGAGGTAATGCATCTTTCTGAGGGGAGAAATGCTGGCCTCTCTCTGGTGCCTTTATTGAGCTGATGTGAGACTCTGGTGCTCACTCAC
AGGCTAGCTTGTCTCCCTTGCTAACCTTGCTGTGGTCCTATCCTCTATCTGAATTGAGAGGTATCTTATCGCTCCCACTCCAGAGAAACTTTAAT
GCTCAGGCTTCAAACTCCCTATCTTCCCTCCTCAGAGGTCCTTCTGTCCCCTTTACTAAAAAAAAAAAAAAGTTCACCCTGCGTCACCAACTGC
CAAAGCATTTTACTCTCCTCTTCTCCCCAAAAGCCTCTTGGAGGAGGGTGGAGCTTAGCCTCAAGAACAAAACTTGGTGCCGGCCAGGTTGAC
ACCTGCACGGGCTGCAGAATTTCCGTACACCTGGAGGCTGAGTACTCTAACGGGCTTTGGAGGATTTTGACATCTTCTATCAAGTTGTCATTTC
CATACACCTGGAGGCTGAGTACTCTAAGGGGCTTTGGAAGATTTTGACATCTTCTATCAAGTTGTCTTCCTTGTGTGTAAATGTGTGTGTTGGG
AGTGGGAAGAGTGTGGGTTGTGGGAGAGGAAGGCAGACTGACAGGCCACCCTTCAGATCACTTAGATCTGAACTCCTTTTTCTGACCTCTTC
TCATCTTTTATAGATAAGGTCAGACTAAAGGATCCTGTCACAGGTCCCTCAAATAGACAGGGAAAACATTAGAGCCATCAGAGGTCGTAACCA
TTTATTTCCAAGTTTCTGAAGCTGTGGGTTTTATGTTGCTTGGTTGGGAGAGGTGAGACGTGAAGACTCGGGTATTGAAATTACTCCTCTACAT
TGGAGTTTACAGGCTGCAGGGGCCTCCCAGGAAGGAACGGGCCTCTCTTGAGTC

PR2:

TTGCTTGGTTGGGAGAGGTGAGACGTGAAGACTCGGGTATTGAAATTACTCCTCTACATTGGAGTTTACAGGCTGCAGGGGCCTCCCAGGAA
GGAACGGGCCTCTCTTGAGTCAGCTCTCTAAGCTCAGGCCTCAGCTATCAGTTCAGCTCAGATCTCAACTGTCACTTAAACCCTTGTGCTTAGA
CTGGGTACCTCTTGCCCTTGCAGATGCTGGATACTTATGGTTGCTCTAATGGTTTCTTTTTGTCTATCTTTGAACCTTTATTCCAGAGATCCAGTT
GTCATCGGTATCCAGGAAGCTCTCCTCTTCCTCCTCCTGACGTCTACCACTACAGTTGCTGGTTGTTGCTAAGGTTGCTGCCATGGTAACATGC
ACATCCTGTTTACACCTTCATCTGGGCAAGTTGGTCTAAGCTAGGAACCTACCTACCCTGGACAACCACTATCATCACCACCTGGGGACACCAA
TCATCGTGACACGGAGTCCACCTTCCACTCAGTTCCCCCATCCTCTTCCTCCTCTCGCTGCCAGACTTCATAGAGAAGAAAGGATCTAGACTTC
GGACGGCTACTCGGGAGCTTATTGCACAAGATATATTCAATCTATTCCCTCACTGGGCCCCCAGAGAAGCAAGAAGTAGGAAGAAGTTGAGA
CAGGGAGGCAGGAGACACTGGTCAGTTGAAGGGAAACGCTACATCTTCTCTGGTTGAGGGGCTTGGTAACAGCAGGCAAA

H1.1:

CTGGTGGGAAAGGTATGGCATCTGAAGCACTGAAAGGAGGGCAGGCCATGAAGAACTGTATCTTTCCCAGAGATGATGTTTAGAAAGTGTG
CTATTCTCTAAAGATTATTCTTTTCTCCTTTTTCTTTTCTTTCTTTCTTCTTCTTTTGTTTCTTTTTTTTTTTTTTTTTTTTTGGTGAGACAGGGTCCT
GCTCTTTTACCCAGGCTGGAGTGCAGTGGCGTGATCATGGCTCACTGCAGCCTCAACCTCCTGGGCTGAAGCCATCTTCCTACCTCAGTCTGCT
GGGTAGCTAGGACCACAGGTGTGTGCCATCACACCCAGCTAGCTTTTTAGATTTTTTTGTAGAGTTGAGGTCTCAGTATCTTGCCTAGACTCAT
CTCAAACTCCTGGACTTAAGTGATTCTTCCACCTTGGCCTCCCAAAGTGTTGGGATTACAGGCACAGTGAGCCACTGCTCCTGACCCCTCTTTT
CTCCTTTTTCTGTATCAGTGTCCAGAAAGGAGGATATTTATTTTTAAAATGTTTATGTTATAAATGTTGTTGTCAGTAAATAATCAAGTCTGTTC
TTCTATATGTCTGATTATAAAATAATTTAATTCTTCCCTACACTGATCTTACAGTTTAATGAATGGAGGAAGGAAGGGTACAAAAAAGTGAATA
AGTCCAGCTGCCC

H1.2:

TGGATCACTCACTGTTTTGTGTCAGGGAATTATAAGTGATTTTTTTTCAACTCTGCTGGATACACTTTTAATGTAGTTCTCCTTAATCTTTTTTGT
TTGTTTTGTTTTTTGTTTTTTTGGTTTTTTTGTTTTGAGAAGGAGTCTCACTCTTGTTGCCCAGGCAGGCTGGAGTGCAATGGTGCAATCTTGGC
TCACTGCAACCTCCACCTCCTGGATTCAAGCAATTATCCTGCTTCAGCCTCCTGAGTAGCTGGGGTTACAGGCATGTGGCTAATTTTGTATTTTT
AGTAGAGATGGAGTTTCACCATTTTGACCACACTGGTCTCAAACTCCTGACCTCAGGTGATCCGCTCACCTCAACCTCCCAAAGTGCTGGGATT
ACAGGTATGAGCCACTGCGCCCGGCCTTAATCTCTTCTTAAAAGCTAAAAAAACTTAAAAGATAATGAAAAATTCAGCTTCAGGCTTTGGCCC
AGCCCAGTTGCCCAGTGTGCCATGGTATTATGAAATTTTGCCTCATCAAAATGTAGAAAGAACAAAAGGAAACTTGCTTTTTCACTTTAGTCTG
GTTTTAGCATCTCTGTAGCAGATTCTTACTTGGCCCTATACATCTGGCAGTTCATGGTTGAGTAGCATGAGATGGAGCAAAGATCTTGTCCATT
AGGGGCTTTCAGTCTCATGTGGTCTCCTATTTCTGAATCTTCCTCAGAGGCCACAGCATATTTCTCACCAATTCTAAGAAGTTGTTACCCTTTGC
CAGTGCTGTATTATAAGTTAGTGACTTGGAGCCTAGACTCTTTAGGATGAAAAAAGAGAAAGAGAGTTTTACATAGATATCTATACCTAGTTT
ATGTCTTATTTTCTTTACAACCTAAGGGAGCTTCCCTACCTGTTTTCTTTACTTCTAGAATTTGGTTTCCTATCTCATAGGCTTTGAGTTCAAATG
ATAGCTCCACCATTTACTAACTAAGTAGCCCTCTACAGTTTACTCAGCCTCCCTGAGTCTGTTTCTTCTTTGAAAATAATGTTAATAACACCTAG
CCCATAGAGTTACTGAGATTAAATAATACATATACAGTGCTCATACTTATAATTAGGTGTTCAATAGATTAGTTTTCTCAGATTAAAACAGAGT
CCATGCATCTGTAGATACTTGCCAGAACATGGGTG

Figure S1. DNA sequences inserted into pGL4-Firefly luciferase plasmid. Location of SNPs of the haplotype highlighted with yellow.

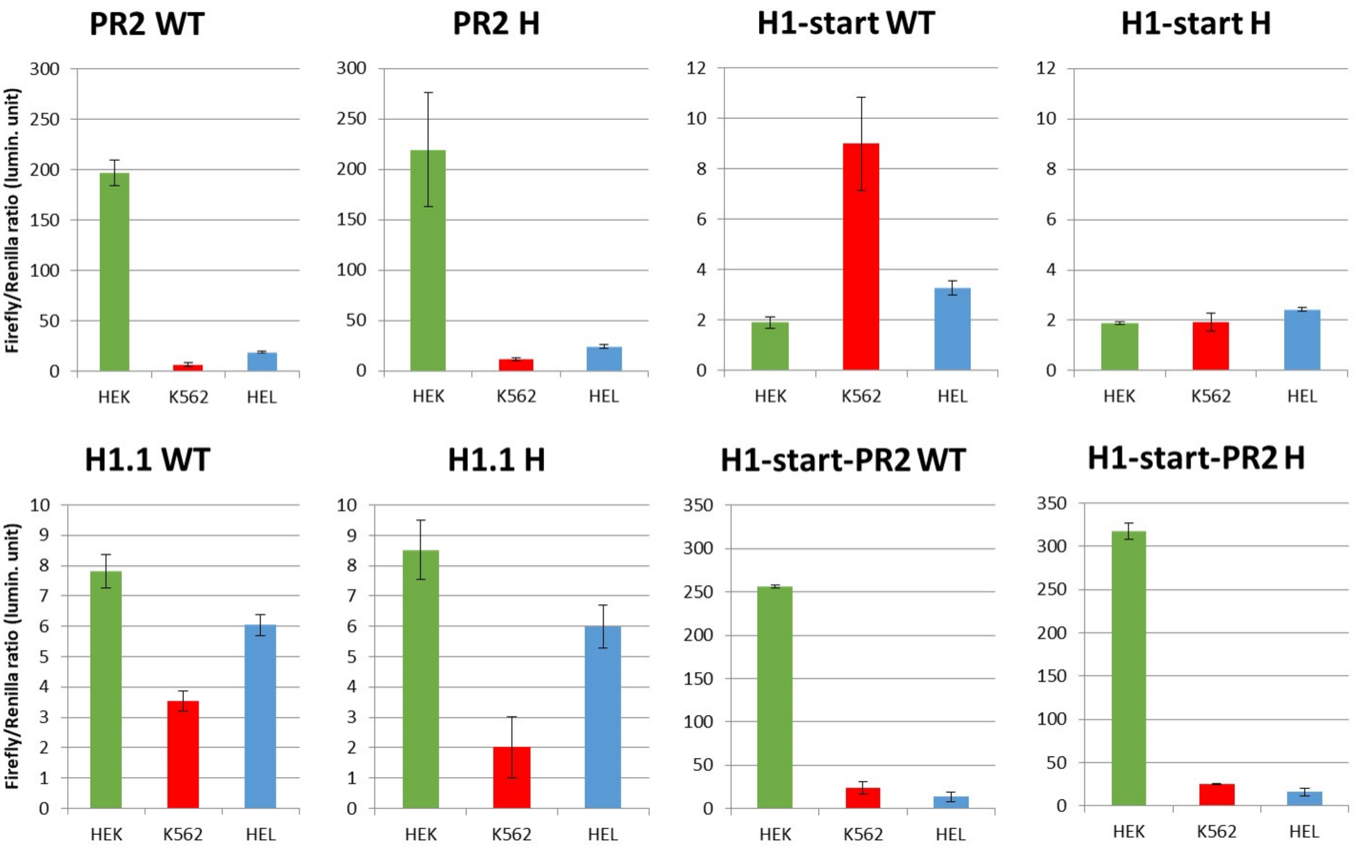

Figure S2: Dual-luciferase measurements with the examined H1st, H1.1, PR2, H1st-PR2 constructs in HEK, HEL92, K562 cells, data visualized prior normalization to HEK WT.
